# Cullings from Contemporaries

**Published:** 1888-08

**Authors:** 


### CULLINGS FROM CONTEMPORARIES.

EDITED BY T. J. BENNETT, M. D.

BICHLORIDE OF MERCURY IN DIPHTHERIA.—Dr. A. Jacobi recommends, in a paper read before the Philadelphia County Medical Society, and published in the Weekly Medical Review of July 14, 1-60th to 1-40th of a grain of the bichloride hourly to children from three to six years of age. Where the nasal cavity is the seat of false membrane, Dr. Jacobi employs one grain of the sublimate and 4 or 5 grains of common salt to the pint of water used as a douche several times a day. Children one year old can take daily one half grain of bichloride of mercury without the drug producing ill effects.

---

TREATMENT OF INFLAMMATIOF IN THE REGION OF THE ILEO-CŒCAL VALVE.—Dr. M. H. Richardson, (Columbus Med. Journal from Boston Med. and Surg. Journal,) in a paper read before the Suffolk District Medical Society, sums up his conclusions as to the proper treatment for these cases in the following propositions:

1. In mild cases of inflammation in the region of the appendix there should be no surgical interference till physical examination reveals the presence of an abscess, which should be incised by the post-peritoneal method.

2. In violent cases, where it is evident that there is a general peritonitis, laparotomy should be done immediately, just as soon as a diagnosis of general peritonitis has been made.

3. In violent cases, where it is doubtful whether the general peritoneal cavity has yet been invaded, and where the history and the physical examination favor the presence of an abscess in the ileo-cœcal region, though it is impossible to locate the exact seat of the inflammation process, an exploratory incision should first be made in the right iliac fossa, and the ileo-cœcal region explored post-peritoneally.

4. The best incision to reach the appendix, in the average case, is along the outer border of the rectus, about four and a half inches from the spine of the pubes.

5. The best incision for extra-peritoneal exploration is parallel with and close to Poupart's ligament, beginning in about the center, and entering outwards and backwards a sufficient distance.

---

DR. GUSTAV A. RENZ, writing from Prague to the Northwestern Lancet for July 15, says Dr. Ruberka has a record of 29 successive cases of vaginal extirpation of the uterus without a single death. These operations were for cancer. Ruberka never undertakes the operation, if the uterus is at all immovable or the vagina infiltrated with cancer.

---

WEIL'S DISEASE.—This is said to be a new disease, discovered by Dr. Weil, in 1886. From a number of medical journals, the following symptoms seem to be agreed upon: It begins suddenly, frequently with a chill, fever following, and continues eight or ten days. Constant symptoms: Fever, headache, gastric disturbance, general muscular pains. Symptoms not constant, but often present: Jaundice, liver tender on pressure and somewhat enlarged, spleen enlarged, kidneys inflamed, and relapses.

This, to a man in the swamp, would sound like malarial fever.

---

HOW TO TREAT CRAMPS IN THE LEG.—It has been suggested by Dr. R. W. St. Clair (Medical Age) that a strong cord wound around the leg and drawn tight over the place that is cramped, will give instant relief. This cures it for the rest of the night. Then Dr. St. Clair advises about six or eight cells of galvanic battery, with negative pole applied over the spot that cramps, and the positive pole over the thigh, as a remedy for permanent relief.

---

IPECACUANHA IN THE TREATMENT OF DISEASE.—Dr. A. S. Holmes, of Benton, Miss., (Memphis Med. Journal) calls attention to the efficacy of ipecac in inturrupting the attacks of intermittent fever. The doctor prescribes the drug in from two to five grain doses every three hours. Dr. Holmes claims almost specific properties for ipecacuanha in the treatment of malarial hæmaturia. The initial dose is ten grains, and if emesis is produced, the dose is reduced to three grains, given at the same interval.

Dr. Holmes makes the assertion that a man can hardly be vomited more than once in six hours with ipecac.

